# Low-Frequency NNRTI-Resistant HIV-1 Variants and Relationship to Mutational Load in Antiretroviral-Naïve Subjects

**DOI:** 10.3390/v6093428

**Published:** 2014-09-16

**Authors:** Shaili Gupta, Max Lataillade, Tassos C. Kyriakides, Jennifer Chiarella, Elizabeth P. St. John, Suzin Webb, Elizabeth A. Moreno, Birgitte B. Simen, Michael J. Kozal

**Affiliations:** 1 Yale University School of Medicine, New Haven, CT 06510, USA; E-Mails: tassos.kyriakides@yale.edu (T.C.K.); jennifer.chiarella@yale.edu (J.C.); Michael.kozal@yale.edu (M.J.K.); 2 Veterans Affairs Connecticut Healthcare System, 950 Campbell Ave, Bldg 1, 5th floor, West Haven, CT 06516, USA; 3 Bristol Myers-Squibb, Global Clinical Research, Wallingford, CT 06492, USA; E-Mail: max.lataillade@bms.com; 4 454 Life Sciences—A Roche Company, Branford, CT 06405, USA; E-Mails: eliz.stjohn@gmail.com (E.P.S.J.); suzin_webb@yahoo.com (S.W.); e.a.moreno86@gmail.com (E.A.M.); bsimen@gmail.com (B.B.S.)

**Keywords:** NNRTI, non-nucleoside reverse transcriptase inhibitors, HIV, resistance, NNRTI resistance mutations, low frequency variants, mutational load, mutant variant frequency

## Abstract

Low-frequency HIV variants possessing resistance mutations against non‑nucleoside reverse transcriptase inhibitors (NNRTI), especially at HIV reverse transcriptase (RT) amino acid (aa) positions K103 and Y181, have been shown to adversely affect treatment response. Therapeutic failure correlates with both the mutant viral variant frequency and the mutational load. We determined the prevalence of NNRTI resistance mutations at several RT aa positions in viruses from 204 antiretroviral (ARV)-naïve HIV-infected individuals using deep sequencing, and examined the relationship between mutant variant frequency and mutational load for those variants. Deep sequencing to ≥0.4% levels found variants with major NNRTI-resistance mutations having a Stanford-HIVdb algorithm value ≥30 for efavirenz and/or nevirapine in 52/204 (25.5%) ARV-naïve HIV-infected persons. Eighteen different major NNRTI mutations were identified at 11 different positions, with the majority of variants being at frequency >1%. The frequency of these variants correlated strongly with the mutational load, but this correlation weakened at low frequencies. Deep sequencing detected additional major NNRTI-resistant viral variants in treatment-naïve HIV-infected individuals. Our study suggests the significance of screening for mutations at all RT aa positions (in addition to K103 and Y181) to estimate the true burden of pre-treatment NNRTI-resistance. An important finding was that variants at low frequency had a wide range of mutational loads (>100-fold) suggesting that frequency alone may underestimate the impact of specific NNRTI-resistant variants. We recommend further evaluation of all low-frequency NNRTI-drug resistant variants with special attention given to the impact of mutational loads of these variants on treatment outcomes.

## 1. Introduction

Standard population sequencing used for genotyping does not detect viral variants that are <20% of the viral population; these viruses are referred to in the literature as “low frequency” or “minority” variants [1]. It is now well established that these low-frequency viral variants can impact clinical outcomes. Many studies have shown that drug resistant variants can persist at very low frequencies, only to re-emerge as the predominant viral population under selective drug pressure and cause therapy failure [1,2,3,4]. Non-nucleoside reverse transcriptase inhibitors (NNRTI) form one of the major classes of antiretroviral (ARV) agents used as standard of care therapy for HIV infection. Specific low-frequency NNRTI-resistant variants (possessing K103N or Y181C) have been strongly associated with virologic failure in subjects initiating NNRTI-based therapy [1,2,3,4,5,6,7,8,9,10,11,12]. This association has been shown to be dose-dependent with increasing mutant variant frequency and quantity (mutational load, *i.e.*, the total number of viral copies of the mutant variant) both having an ascending risk of virologic failure [12]. Few studies have examined the relationship between mutant variant frequency and mutational load for all NNRTI-resistance mutations that may be identified in ARV-naïve HIV-infected individuals. Mutations at several aa positions on the reverse transcriptase (RT) gene are known to confer high level resistance to efavirenz (EFV) and nevirapine (NVP), many of which also cause resistance to etravirine (ETR) and rilpivirine (RPV) and increase the risk of treatment failure. We determined the prevalence of HIV variants possessing NNRTI-resistance mutations among ARV-naïve individuals and sought to ascertain the relationship between mutant variant frequency and mutational load. 

## 2. Materials and Methods

De-identified plasma samples prior to initiation of ARV therapy were collected from ARV-naïve HIV-infected subjects enrolled between 2005 and 2010 in two prospective randomized controlled trials (CASTLE: Comparison of atazanavir/ritonavir* versus* lopinavir/ritonavir in combination with tenofovir-emtricitabine to assess safety and efficacy in antiretroviral-naïve subjects; SPARTAN: Nucleoside- and ritonavir-sparing regimen containing atazanavir plus raltegravir in antiretroviral treatment-naïve) [13,14] and from Yale HIV Sample Resistance Databank (a repository of HIV-infected plasma samples collected for use in various studies). The study was approved by Human Research Protection Programs at all sites involved. All participants provided written informed consent. Subjects’ samples were from five continents with the majority from United States and Europe. HIV subtypes were found to be from A, AE, B, BF, C, F and some CRF subtypes, the majority being subtype B. Baseline HIV viral loads were determined on these plasma samples. HIV RNA was extracted and deep sequencing (454 Life Sciences-Roche, Branford, CT, USA) was performed as described previously [1,14,15]. Samples were evaluated for low-frequency variants possessing NNRTI resistance mutations. Major NNRTI-resistance mutations were defined as having a Stanford-HIVdb algorithm value ≥30 indicating intermediate to high-level resistance to efavirenz (EFV) and/or nevirapine (NVP) [16]. These include mutations on RT gene at aa positions L100, K101, K103, V106, E138, V179, Y181, Y188, G190, P225, F227, M230 and K238. An estimated mutational load was calculated by multiplying variant frequency by HIV RNA copies/mL. In subjects who had >1 variant with major NNRTI mutations, the mutational load was calculated by multiplying the highest variant frequency by HIV RNA copies/mL.

Minority variants were detected to a lower limit of 0.4% in all subjects, and to an additional limit of 0.2% in 56 of the 204 subjects because of improvement in the technique over time, permitting us to apply that new limit to samples processed later. In these 56 subjects, we also looked for mutations at position E138 and V179, which had been newly identified as relevant sites for mutations conferring resistance to rilpivirine (RPV). 

## 3. Statistical Methods

Data on mutations were analyzed both at the patient level and mutation level. Descriptive statistics (Mean; Median, Interquartile Range (IQR)) are provided for continuous variables. Frequency distributions are also provided for categorical variables. Parametric or non-parametric techniques were used as appropriate to compare continuous variables. Chi-square methods were used to assess the association between categorical variables. Pearson correlation analysis was also carried out between mutant variant frequency (% level) and mutational load or log mutational load. 

Linear regression analysis was used to analyze mutational load (dependent variable) and mutant variant frequency level data; regression plots with 95% Confidence Interval (CI) bands around the regression-fitted line were generated and displayed. All analyses were carried out using SAS/STAT software [17]. 

## 4. Results 

Two hundred and four ARV-naïve subjects were evaluated by deep sequencing for NNRTI-resistant variants. Mean viral load at baseline was 201,402 (Median 111,500; IQR 37,250–277,500) copies/mL. Fifty-two (25.5%) subjects had major NNRTI-resistant (Stanford score ≥30 against EFV or NVP) variants detected at frequency ≥0.4%, of whom 14 (6.9%) possessed multiple NNRTI-mutations (Table 1). 

**Table 1:** Summary of mutant variants with frequency prevalence ≥0.4%.

Mutant Variant	Number Detected	Number of Patient Samples Tested	% Patient Samples with Mutant Variant	Range of Mutant Variant Frequency (%)	Range of Mutational Load (copies/mL)
L100I	2	204	1.0	0.41–0.42	638–2005
K101E	6	204	2.9	0.48–3.28	76–8100
K103N	17	204	8.3	0.50–67.60	163–368,420
K103S	2	204	1.0	2.50–36.50	2483–28,835
K103T	2	204	1.0	1.09–2.79	2204–3074
V106A	1	204	0.5	0.40	182
E138A *	2	56	3.6	1.37–1.60	3632–9754
E138K *	2	56	3.6	0.48–0.51	571–811
V179D *	1	56	1.8	4.07	1400
V179E *	3	56	5.4	0.58–97.92	3074–142,963
Y181C	10	204	4.9	0.44–98.80	168–741,001
Y181I	2	204	1.0	1.67–96.46	558–354,008
Y188C	1	204	0.5	1.49	124
Y188H	3	204	1.5	0.40–0.93	75–972
G190A	4	204	2.0	0.44–4.57	717–34,275
G190E	7	204	3.4	0.40–1.29	25–3300
P225H	6	204	2.9	0.49–18.33	307–7124
K238N	1	204	0.5	0.43	439

* SPARTAN specimens were tested for mutant variants at position 138 and 179. Of 204 subjects, 52 were found to harbor a total of 72 major Non-nucleoside reverse transcriptase inhibitor (NNRTI)-resistance mutations at a frequency of 0.4% or above.

**Figure 1:**
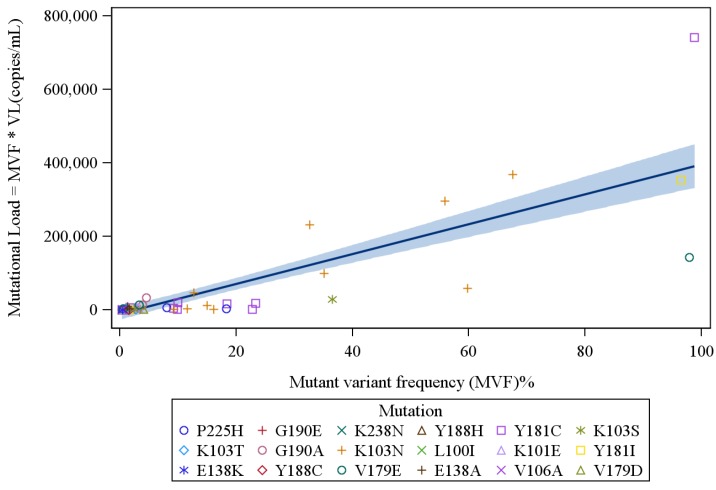
Mutational-level analysis of all mutations found, Frequency greater or equal to 0.4%: A total of 72 NNRTI mutations were identified in 52 ARV-naïve HIV-infected subjects. The NNRTI mutant variant frequency (MVF) and mutational load were highly correlated (Spearman’s correlation 0.83; *p* < 0.0001). [VL= Viral load *i.e.*, Total HIV RNA copies/mL detected in subject’s plasma].

**Figure 2:**
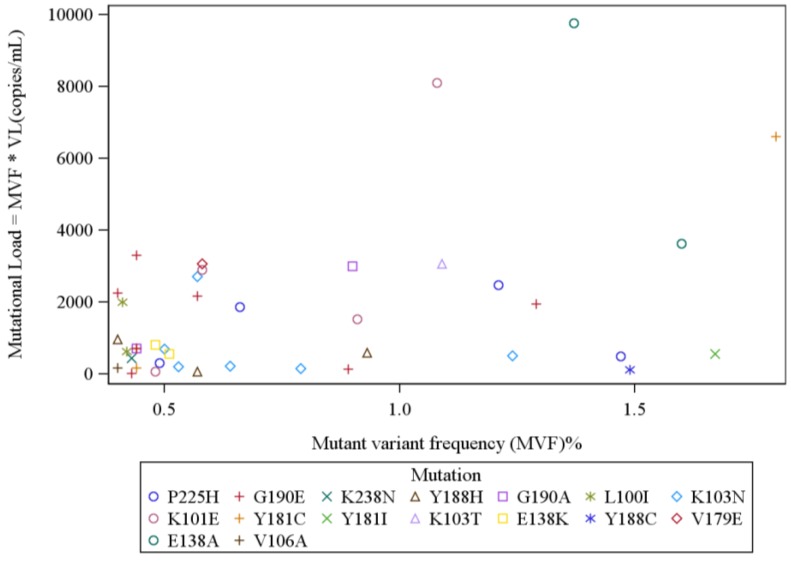
Mutational-level analysis of mutations detected at frequency 0.4% to 2%: The correlation between mutational load and mutant variant frequency (MVF) was weaker at lower MVFs. (Spearman’s correlation 0.49, *p* = 0.0002). [VL = Viral load *i.e.*, Total HIV RNA copies/mL detected in subject’s plasma].

The mean baseline HIV RNA level trended higher at 227,361 (Median = 142,500; IQR = 38,000–374,500) copies/mL among the 52 subjects with detectable low-frequency variants with major NNRTI mutations, as compared to 192,522 (Median = 107,000; IQR = 36,500–245,000) copies/mL among the 152 without such variants (*p* = 0.32 *i.e.*, not suggestive of a significant trend). Among these 52 subjects, a total of 72 major NNRTI-resistance mutations were found at a frequency of 0.4% or above. K103 and Y181 were the commonest aa positions with detectable mutations, followed by aa positions G190, K101 and P225 (Table 1). A majority of these variants (39 variants) were found at a frequency >1% and/or were accompanied by multiple resistance mutations in the same subject. The frequency of NNRTI-resistant variants ranged from 0.4%–98.8% (mean 11.5%, median 1.42%; IQR 0.55–9.97). The median variant mutational load was 2094 (mean 47,664; IQR 535–11,114) copies/mL. Overall, mutant variant frequency and mutational load for the 72 NNRTI mutations identified were highly correlated (Spearman’s correlation 0.83; *p* < 0.0001) (Figure 1). The correlation between log mutational load and variant frequency was also significant (Spearman’s correlation 0.74; *p* < 0.0001). However, the range of mutational loads for variants at very low-frequency was large; e.g., variants at frequency <2% (46 subjects with total 53 mutations) had a median mutational load of 564 (mean 1531; IQR 181.6–2256) copies/mL; with a correlation between variant frequency and mutational load less pronounced (Spearman’s correlation 0.49, *p* = 0.0002) (Figure 2). The correlation between variant frequency and the log mutational load was also less significant for these very low frequency variants (Spearman’s correlation 0.41, *p* = 0.002). 

Among the 56 subjects from SPARTAN study who had exploration for additional mutations at positions E138 and V179, 15 were found to have 20 relevant mutations at 0.4% or above, five of whom had variants with multiple mutations. Mean viral load was 213,616 (median 162,000; IQR 45,400–282,000) copies/mL. The range of frequency of these 20 mutations varied from 0.4%–97.9%, with a median frequency of 1.55%, IQR 0.55%–18.3%. The mean mutational load for these 20 mutations was 43,845 (median 3074; IQR 891.6–36,740) copies/mL; Spearman’s correlation 0.73, *p* = 0.0003. In these 56 subjects from SPARTAN study, we performed deep sequencing to a limit of 0.2% because of improved technology available when the samples were processed. A total of 24 subjects were found to have variants with 32 mutations when the lower limit of detection was extended to 0.2% (Mean viral load 180,939 copies/mL; median 135,500 copies/mL; IQR 49,500–235,000 copies/mL). The range of frequency of these 32 mutations varied from 0.22%–97.92%, with a median frequency of 0.5%, IQR 0.26%–2.9%. The mean mutational load for these 32 mutations was 27,525 (median 758.7; IQR 257.8–4472.8) copies/mL; Spearman’s correlation 0.75, *p* < 0.0001. Mutations with all scores at positions E138 and V179 were included in these 32 mutations as a single amino-acid change could result in major mutations causing RPV resistance. Of the 56 SPARTAN study subjects, nine were found to have variants with mutations at position E138 (five subjects with a total of 16 mutations) and position V179 (four subjects with a total of four mutations). 

**Table 2:** Summary of subtypes and non-nucleoside reverse transcriptase inhibitor (NNRTI) mutant variants with prevalence ≥0.4%.

Subtype	Subjects from Each Subtype	Unique Subjects with NNRTI Mutations	NNRTI Mutations	Range of Frequency Prevalence ≥0.4%
A	1	0		
AE	7	2	K101E, K103N, Y181C, P225H	0.58–18.44
B	144	38	L100I, K101E, K103N/S/T, E138A/K, V106A, V179D/E Y181C/I, P225H, G190E, K238N, Y188C/H, G190A/E, P225H	0.4–97.92
BF	18	7	K101E, K103N, Y181C, Y188H, G190E	0.5–98.8
C	27	5	K103N, Y181C, G190A P225H	0.44–9.3
CRFs	5	0		
F	2	0		
Total	204	52		

HIV subtypes in plasma samples from 204 subjects were found to be from varied subtypes, the majority being from subtype B. The table enlists distribution of various NNRTI-resistance mutations and their frequencies across different subtypes.

At frequency ≥0.4%, 18 different mutations were identified at 11 major NNRTI-resistance sites (total 72 mutations found) in 52 total subject samples (see Table 1). Mutations at each RT site are listed with the total number (n) detected at frequency ≥0.4%, followed by the percent of subjects who tested positive for those mutations, to give an idea of transmitted NNRTI resistance (Percentage calculations for most mutations are done off 204 study samples, except for positions E138 and V179, which were analyzed in only 56 samples as these RT positions were not evaluated in the deep sequencing studies prior to 2012): K103N/S/T (n = 21, 10.3%), Y181C/I (n = 12, 5.9%), G190A/E (n = 11, 5.4%), K101E (n = 6, 2.9%), P225H (n = 6, 2.9%), E138A/ K (n = 4, 7.1%), V179D/E (n = 4, 7.1%), Y188H/C (n = 4, 2%), L100I (n = 2, 0.1%), V106A (n = 1, 0.5%) and K238N (n = 1, 0.5%) (Table 1). No mutations were detected at the aa positions F227 and M230 among the study subjects. Distribution of NNRTI-resistance mutations according to HIV subtypes is provided in Table 2. 

At frequency ≥0.2%, additional variants were detected, e.g., E138K, a major resistance mutation against RPV, was found in four subjects (frequency 0.32%–0.51%; mutational load median = 374 copies/mL (IQR = 162–691 copies/mL), and 10 subjects had E138G (frequency 0.22%–0.38%; mutational load median = 320 copies/mL (IQR = 258–480 copies/mL). 

## 5. Discussion

In this study, low-frequency variants possessing major NNRTI-resistance mutations were identified in 25.5% of ARV-naïve HIV-infected subjects. The identification of 18 different major NNRTI-resistant mutations suggests the need to screen for more than just K103N and Y181C prior to initiation of NNRTI-based therapy in ARV-naïve individuals. K103N and Y181C were the commonest mutations detected, along with G190E, K101E and P225H. In addition, many mutations were detected, including other major mutations on positions K103 and Y181. A majority of these variants (39 variants) were found at a frequency >1% and/or were accompanied by multiple resistance mutations in the same subject, raising the likelihood that these represented transmitted ARV resistance. For minority variants at <1% frequency, sources other than transmission may be relevant, including viral errors introduced during HIV viral replication and recombination, or assay errors during RT-PCR amplification and sequencing [15,18]. 

In this study, a strong correlation between NNRTI-resistant variant frequency and mutational load was identified; however, variants at low frequency had a wide range of mutational loads (>100-fold) suggesting that frequency alone may underestimate the impact of specific NNRTI-resistant variants, a very important finding. 

Our study had some limitations as follows. The mutational load was calculated by multiplying results from two separate molecular assays—baseline HIV viral load and mutant variant frequency by deep sequencing—and thus should only be considered an estimate as different primers are used to generate the RT-PCR amplicons from HIV RNA templates for the two assays which can lead to sampling bias. The frequency of variants at very low levels may or may not be truly reflective of the proportion of the viral quasispecies given the limitations of viral RNA extraction techniques [15]. However, the majority of subjects had baseline HIV viral loads >100,000 copies/mL, suggesting that most specimens provided a representative sample of HIV variants present in plasma [1,12]. Our study was not designed to correlate the findings of deep sequencing to virologic outcomes because all sequencing was performed on de-identified plasma samples with no linking information on the clinical outcome of these patients, except the information that all patients went on to Protease inhibitor based antiretroviral therapy. Therefore, we cannot comment on the clinical relevance of the mutations detected in this study. E138K, the major mutation for RPV resistance, was noted in only five subjects at frequencies below 1% and at mutational loads well below 2000 copies/mL. Three of these five subjects with E138K did not have any concomitant NNRTI mutations and therefore assay errors cannot be ruled out. Conversely, E138K may represent a natural polymorphism albeit at low frequency. 

Although we cannot correlate the frequency and mutational load to virologic outcome, as this was not the goal of the study, Li and colleagues [12] reported a strong dose-dependent effect of mutational load on virologic failure. Mutational load becomes especially relevant in those with high viremia (>100,000 copies/mL) at baseline, and the quantitative as well as qualitative reliability of HIV RNA extraction and deep sequencing reads increases proportionally with the number of HIV RNA copies/mL [15]. High mutational load variants even though present at very low frequency could raise the potential for an adverse effect on clinical outcome. Such impact is most relevant for NNRTIs, as they are known to have a low genetic barrier to the development of HIV resistance, and have also been shown to have higher risk of virologic failure with baseline high viremia [19,20]. This is especially true for the newest NNRTI, rilpivirine, which was shown to have higher risk of virologic failure with high baseline VL and higher number of treatment-emergent NNRTI-resistance mutations, most commonly E138K and K101E [21]. In our study, a limited number of subject samples underwent investigation for presence of E138K, which, when found, was low in frequency and mutational load. 

The clinical relevance of very low frequency variants is under intense investigation. Our finding that a significant portion of ARV-naïve subjects possess low frequency variants, totaling 18 different NNRTI mutations, many at high mutational loads, suggests that future studies should screen for all NNRTI mutations and not just a few sentinel mutations (e.g., K103N and Y181C) when investigating the impact of NNRTI minority variants. In addition, the impact of mutational load should be included in such investigations, not just mutant frequency, as the absolute quantity of mutant variant can be quite different at similar frequencies. 
